# CCL5-dependent mast cell infiltration into the tumor microenvironment in clear cell renal cell carcinoma patients

**DOI:** 10.18632/aging.103999

**Published:** 2020-11-11

**Authors:** Tianjie Liu, Qing Xia, Haibao Zhang, Zixi Wang, Wenjie Yang, Xiaoyun Gu, Tao Hou, Yule Chen, Xinqi Pei, Guodong Zhu, Dalin He, Lei Li, Shan Xu

**Affiliations:** 1 Department of Urology, The First Affiliated Hospital of Xi’an Jiaotong University, Xi’an 710061, Shaanxi, P.R. China; 2 Oncology Research Laboratory, Key Laboratory of Environment and Genes Related to Diseases, Ministry of Education, Xi’an 710061, Shaanxi, P.R. China; 3 Key Laboratory for Tumor Precision Medicine of Shaanxi Province, Xi’an Jiaotong University, Xi’an 710061, Shaanxi, P.R. China; 4 Department of Oncology, State Key Laboratory for Oncogenes and Related Genes, Renji Hospital, School of Medicine, Shanghai Jiaotong University, Shanghai Cancer Institute, Shanghai 200127, P.R. China; 5 Shaanxi Health Information Center, Health Commission of Shaanxi Province, Xi’an 710061, Shaanxi, P.R. China

**Keywords:** RCC, PBRM1, CCL5, tumor microenvironment, mast cell

## Abstract

We investigated the mechanisms affecting tumor progression and survival outcomes in *Polybromo-1*-mutated (*PBRM1*^MUT^) clear cell renal cell carcinoma (ccRCC) patients. *PBRM1*^MUT^ ccRCC tissues contained higher numbers of mast cells and lower numbers of CD8^+^ and CD4^+^ T cells than tissues from *PBRM1*^WT^ ccRCC patients. Hierarchical clustering, pathway enrichment and GSEA analyses demonstrated that *PBRM1* mutations promote tumor progression by activating hypoxia inducible factor (HIF)-related signaling pathways and increasing expression of vascular endothelial growth factor family genes. *PBRM1*^MUT^ ccRCC tissues also show increased expression of C-C motif chemokine ligand 5 (CCL5). PBRM1-silenced ccRCC cells exhibited greater Matrigel tube formation and cell proliferation than controls. In addition, HMC-1 human mast cells exhibited CCL5-dependent *in vitro* migration on Transwell plates. High CCL5 expression in *PBRM1*^MUT^ ccRCC patients correlated with increased expression of genes encoding IFN-γ, IFN-α, IL-6, JAK-STAT3, TNF-α, and NF-ΚB. Moreover, high CCL5 expression was associated with poorer survival outcomes in ccRCC patients. These findings demonstrate that CCL5-dependent mast cell infiltration promotes immunosuppression within the tumor microenvironment, resulting in tumor progression and adverse survival outcomes in *PBRM1*^MUT^ ccRCC patients.

## INTRODUCTION

According to the World Health Organization statistics in 2018, the incidence and mortality rates of kidney cancer were 4.5% and 2.0%, respectively, with 403,262 newly diagnosed cases and 175,098 deaths reported worldwide [1]. Clear-cell renal cell carcinoma (ccRCC) accounts for approximately 75% of all RCC cases [2]. The most commonly mutated gene in ccRCC is *VHL* (Von Hippel-Lindau), which encodes a tumor suppressor protein [2, 3]. Functional loss of *VHL* stabilizes the hypoxia-inducible factor (HIF) and activates the VEGF (vascular endothelial growth factor) and mTOR (mammalian target of rapamycin) signaling pathways [3, 4]. The standard therapy for ccRCC patients includes tyrosine kinase inhibitors targeting the VEGF signaling pathway, such as sunitinib and pazopanib, and mTOR kinase inhibitors, such as, everolimus and temsiromus [5, 6]. However, disease relapse is common in ccRCC patients treated with tyrosine kinase inhibitors or mTOR inhibitors [3]. Recent clinical trials with immune checkpoint inhibitors such as nivolumab and ipimumab demonstrate improved safety and robust antitumor activity in ccRCC patients [7–10]. Furthermore, differential clinical responses to treatment with tyrosine kinase inhibitors and immune checkpoint inhibitors suggest that *VHL*-independent mechanisms may influence tumor progression in ccRCC.

*PBRM1* (Polybromo-1) gene is located on chromosome 3p21 and is the second most frequently mutated gene in ccRCC [11]. *PBRM1* encodes a subunit of the nucleosome remodeling complex called polybromo-1 (PBRM1), also called as BAF180 or BRG1-associated factor 180 [12]. *PBRM1* mutations that disrupt the nucleosome remodeling complex have been implicated in RCC, non-small cell lung cancer, and prostate cancer [12–16]. As far as we known, there is no consistent conclusion about PBRM1 mutations/PBRM1 low expression with ccRCC prognosis and immunotherapy response. In Kapur et al’s. report, limiting the sample size, follow-up, and patient populations, there was no conclusion whether PBRM1 are independent predictors of outcome in ccRCC [17]. In Hakimi et al’s. report, PBRM1 mutations also did not impact cancer-specific survival [18]. However, there were opposite reports claiming that loss of PBRM1 is associated with advanced tumor stage, low differentiation grade tumors, and worse patient survival outcomes [19–22]. The different results indicated the function of PBRM1 protein in ccRCC need further study. Moving forward, ccRCC tumors with *PBRM1* mutations are associated with higher expression of angiogenetic genes [23]. *PBRM1* mutations also correlate with outcomes in ccRCC patients treated with immune checkpoint inhibitors [24, 25]. However, there is extensive literature indicating the contrary. Xian-De et al. reported that *PBRM1* mutations were associated with poor response to immune clinical response therapy in nearly 700 ccRCC patients [26]. However, Miao et al. reported that *PBRM1* mutations were associated with better immune clinical response therapy in more than 100 ccRCC patients [25], and also in David et al’s. report, they revealed that PBRM1 mutations were associated with improved response, progression free survival and overall survival with PD-1 blockade in 592 patients with advanced ccRCC cohort [27]. Immune clinical response was affected by immune tumor microenvironment, but the mechanisms by which mutations in *PBRM1* modulate the tumor microenvironment (TME) are still poorly understood, which need further study.

The TME includes fibroblasts, pericytes, endothelial cells, and immune cells such as T cells, mast cells, and macrophages [28–30]. Mast cells are one of the earliest cell types that infiltrate developing tumors [31]. They secrete several pro-angiogenic factors such as VEGF, basic fibroblast growth factor (bFGF), angiopoietin-1 (ANG-1), heparin, and tumor necrosis factor alpha or TNF-α [32]. They also secrete or express several chemokines and cytokines that modulate immune function such as interleukin 5 (IL-5), IL-6, MHC II (major histocompatibility complex, class II), and TNF-α [32, 33]. In ccRCC tissues, higher numbers of mast cells correlate with increased microvascular density [34–37]. Furthermore, mast cells, ccRCC cells, and endothelial cells interact via the SCF (stem cell factor)/c-Kit signaling pathway [38]. In ccRCC tissues, the status of *VHL* mutations do not correlate with the expression of immune cells [25], whereas, *PBRM1* mutations are associated with T cell infiltration and immune-related gene expression [25]. However, the mechanistic details of the crosstalk between *PBRM1* mutations in ccRCC cells, the tumor microenvironment, and immune cell infiltration and function is not clear.

In this study, we investigated mechanisms through which PBRM1-mutated (PBRM1MUT) ccRCC cells modulate the tumor micro-environment and tumor-infiltration of immune cells using gene expression data from ccRCC patients in the TCGA database and in vitro experiments using ccRCC cell lines.

## RESULTS

### PBRM1^MUT^ patients exhibit altered immune cell profiles in the tumor microenvironment

**Figure 1:**
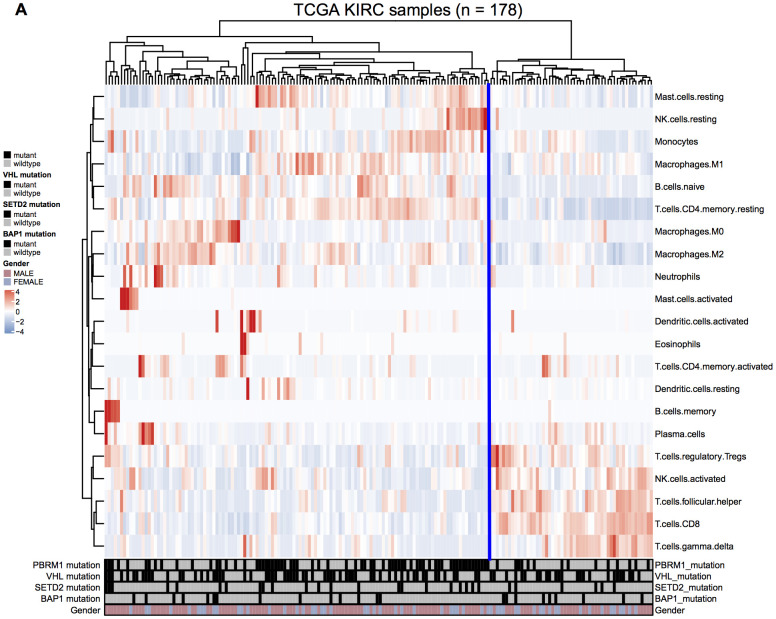
**Immune cell landscape of ccRCC samples.** (**A**) The distributions of twenty-two different immune cell types in the 178 ccRCC samples from the TCGA KIRC database are shown.

We analyzed the gene expression and mutation data of 178 ccRCC patients in the TCGA KIRC database to evaluate the relationship between mutations in *VHL*, *PBRM1*, *BAP1*, and *SETD2* genes in the ccRCC tissues and the infiltration of 22 different immune cell types in the TME. We observed that *VHL* and *PBRM1* were mutated in 47% and 40% of ccRCC patients (Supplementary Figure 1A). Among the 21 immune cell subpopulations (naïve CD4^+^- T cells were excluded), we observed higher proportions of resting mast cells and reduced numbers of resting memory CD4^+^ T cells, M2 macrophages, CD8^+^T cells, activated NK cells, and regulatory T cells and other immune cell types (Figure 1). Furthermore, analysis of immune cell profiles of ccRCC patients suggested immune suppression in PBRM1^MUT^ ccRCC patients (Supplementary Figure 1B). These results show that *PBRM1* mutation in the ccRCC cells promotes immune suppression and alters immune cell profiles in the TME.

### PBRM1^MUT^ tissues show increased tumor purity and mast cell infiltration in the TME

**Figure 2:**
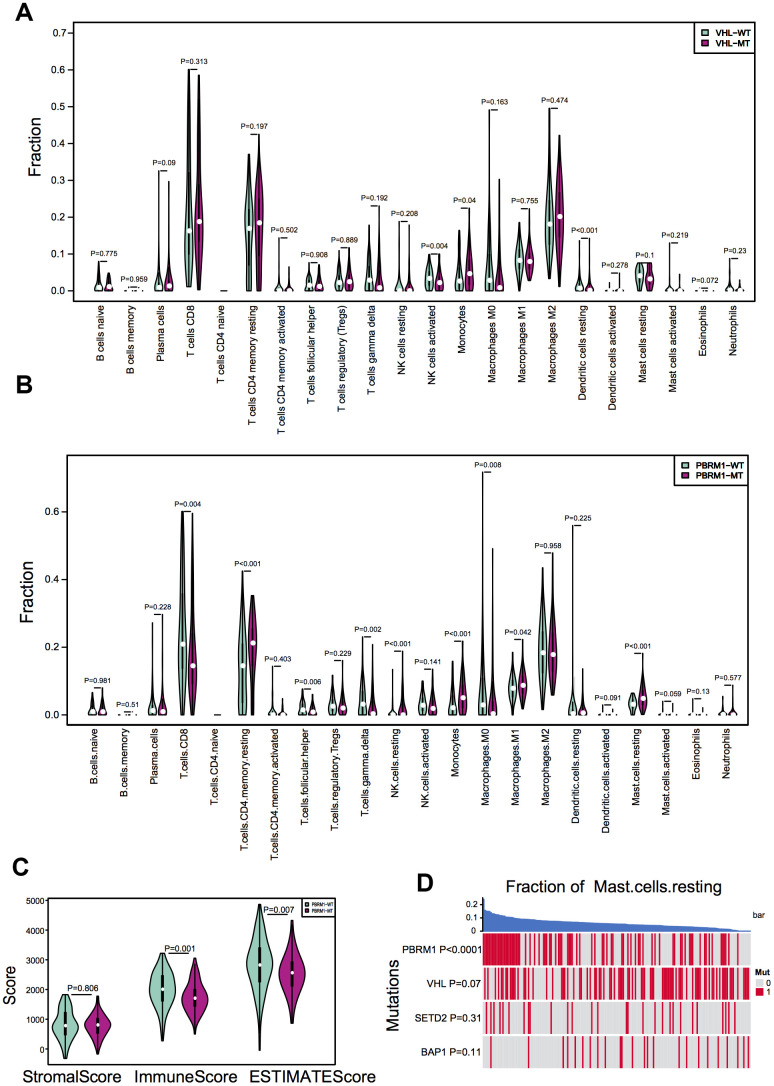
***PBRM1* mutations alter immune cell infiltration patterns in ccRCC samples.** (**A**) Violin plots show different patterns of immune cell infiltration patterns in VHL^WT^ (90) and VHL^MUT^ (94) patients from the TCGA KIRC database. (**B**) Violin plots show different immune cell infiltration patterns in PBRM1^WT^ (97) and PBRM1^MUT^ (81) patients from the TCGA KIRC database. (**C**) Violin plots show stromal and immune purity scores from the ESTIMATE algorithm analyses for PBRM1^WT^ and PBRM1^MUT^ patients in the TCGA KIRC database. (**D**) Heatmap shows the mast cell infiltration status in *PBRM1*^MUT^, *VHL* ^MUT^, *SETD2* ^MUT^ and *BAP1* ^MUT^ ccRCC patients from the TCGA KIRC database.

We next evaluated the effects of mutations in *VHL* and *PBRM1* genes on the infiltration of 22 different immune cell subpopulations in ccRCC tissues. The violin plots show the differences in the proportions of different immune cell populations in the *VHL*^WT^ and *VHL*^MUT^ as well as *PBRM1*^WT^ and *PBRM1*^MUT^ ccRCC patients (Figure 2A, 2B). The sample dendrograms are shown in Supplementary Figure 2 and Supplementary Figure 3. Our results observed that the proportion of different immune cell types was similar in VHL^MUT^ and VHL^WT^ ccRCC tumors (Figure 2A). However, PBRM1^MUT^ ccRCC tissues showed lower proportions of CD8^+^ T cells (P=0.004) and activated CD4^+^ memory T cells (P=0.403), and significantly higher proportion of resting mast cells (P<0.001) compared to the PBRM1^WT^ ccRCC tissues (Figure 2B), and other immune cell types in the ccRCC TME exhibited weak correlation with the PBRM1^MUT^ genotype.

The comprehensive understanding of infiltrating stromal and immune cells in tumour tissue not only perturb the tumour signal in molecular studies but also have an important role in cancer biology [39]. We assessed infiltrating stromal and immune cells in tumor tissues to predict tumor purity in PBRM1^MUT^ ccRCC patients. As shown in Figure 2C, the PBRM1^MUT^ ccRCC patients showed significantly lower immune scores (P=0.001) and ESTIMATE scores (P=0.007) based on ESTIMATE algorithm analyses compared to PBRM1^WT^ ccRCC patients. Furthermore, resting mast cell recruitment positively correlated with the PBRM1^MUT^ genotype (P<0.0001), but showed no significant correlation with mutations in the *VHL* (P=0.0731), *SETD2* (SET domain containing 2; P=0.306), and *BAP1* (BRCA1 associated protein 1; P=0.106) genes (Figure 2D). These results demonstrate that tumor purity and recruitment of resting mast cells to the TME is significantly higher in PBRM1^MUT^ ccRCC patients compared to PBRM1^WT^, VHL^MUT^, SETD2^MUT^ and BAP1^MUT^ ccRCC patients.

### *PBRM1* mutated ccRCC tumors show higher infiltration of mast cells and activation of cell growth and tumor angiogenesis pathways

**Figure 3:**
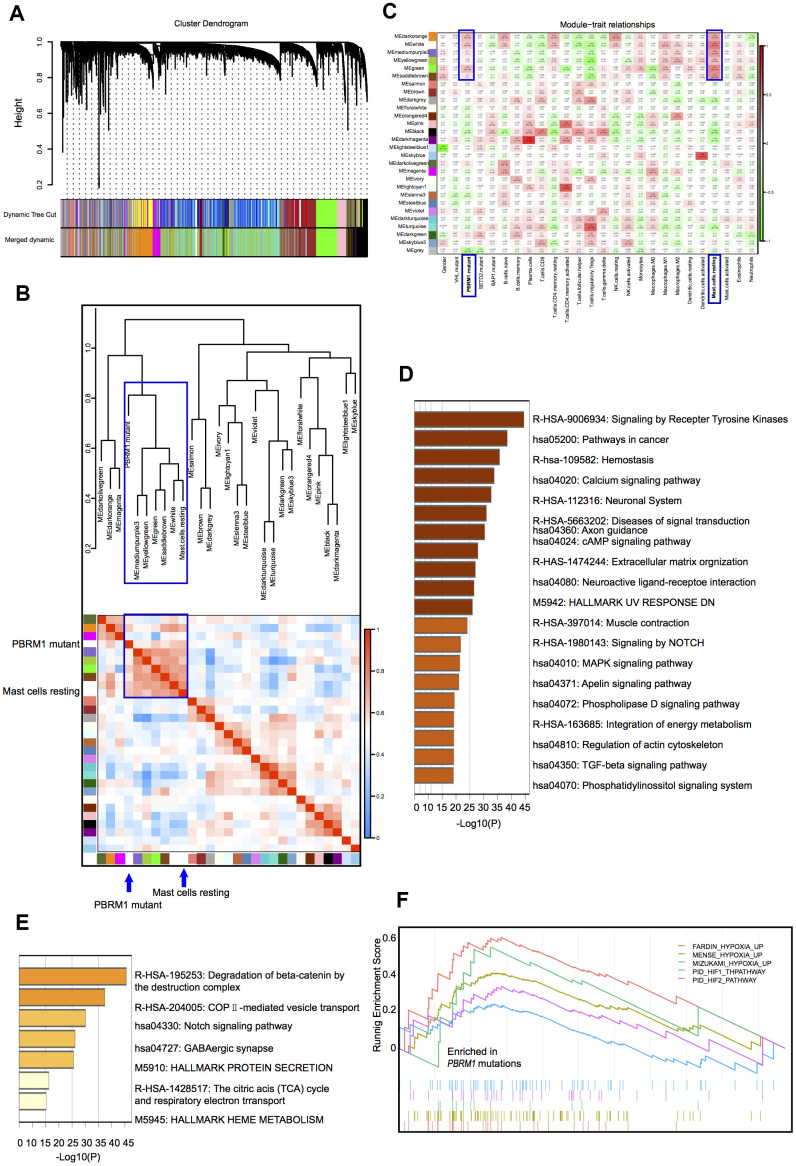
**Correlation analyses between gene cluster modules, *PBRM1* mutations, and mast cell infiltration in ccRCC patients.** (**A**) The clustering dendrogram shows different gene cluster modules that are color-coded. The dissimilarity of genes is based on the topological overlap. (**B**) Heatmap shows the correlation between module eigengenes and immune cell infiltration in ccRCC samples. The correlation table is color-coded. The modules in the blue box are associated with *PBRM1* mutations and mast cell infiltration. (**C**) Analysis of the association between the 27 gene cluster modules and the 4 mutant genotypes (*VHL*, *PBRM1*, *SETD2*, and *BAP1*) in ccRCC patients. Each cell represents a module correlation co-efficient and its corresponding p-value. (**D**) Pathway enrichment analysis of dark orange module. Dark orange gene cluster was positive with *PBRM1* mutant and mast cell infiltration. (**E**) Pathway enrichment analysis of the white module. White gene cluster was positive with *PBRM1* mutant and mast cell infiltration. (**F**) Enrichment plots show upregulated FARDIN hypoxia signaling (red), MENSE hypoxia signaling (green), MIZUKAMI hypoxia signaling (green), PID-HIF1-THPATHWAY (purple), PID-HIF2-PATHWAY (blue), and other gene sets in the PBRM1^mut^ group of ccRCC patients. FARDIN hypoxia signaling gene set including the genes in the hypoxia signature, based on analysis of 11 neuroblastoma cell lines in hypoxia and normal oxygen conditions; MENSE hypoxia signaling gene set including hypoxia response genes up-regulated in both astrocytes and HeLa cell line; MIZUKAMI hypoxia signaling gene set including the genes up-regulated in colon cancer cells in response to hypoxia, might not be direct targets of HIF 1α; PID-HIF1-THPATHWAY gene set including the gens in HIF 1α transcription factor network; PID-HIF2-PATHWAY gene set including the gens in HIF 2α transcription factor network.

Hierarchical clustering analysis of the gene expression data of ccRCC samples in the TCGA database identified 28 gene modules (Figure 3A). This included two large modules (dark orange and green), 25 small modules, and another gray module that contains non-clustering genes. We then assessed the relationship between the gene cluster modules, immune cell types, and the mutant *PBRM1* genotypes in ccRCC patients, we observed strong correlation between PBRM1^MUT^ and the numbers of resting mast cells (Figure 3B). Analysis of the association between 27 gene cluster modules and 4 mutant genotypes (*VHL*, *PBRM1*, *SETD2*, and *BAP1*) in ccRCC patients showed strong correlation between six gene modules (dark-orange, white, medium purple3, yellow-green, green, and saddlebrown). Further verified that mast cell infiltration was positive with PBRM1^MUT^, not *VHL*, *SETD2*, and *BAP1* mutation, respectively (Figure 3C). We also observed three modules (dark-orange, white, and green) were significantly with PBRM1^MUT^ and mast cell infiltration (P<0.001, P<0.001, and P<0.001, respectively). Pathway analysis of the dark-orange and white modules showed that they contained genes played a role in the Notch, MAPK (mitogen-activated protein kinase), and TGF-β (transforming growth factor-β) signaling pathways, tyrosine kinases, and extracellular matrix organization (Figure 3D, 3E), this suggests that *PBRM1*^MUT^ enhanced ccRCC progression though the Notch, MAPK, and TGF-β pathways. Furthermore, GSEA showed activation of FARDIN hypoxia signaling (red), MENSE hypoxia signaling (green), MIZUKAMI hypoxia signaling (green), PID-HIF1-THPATHWAY (purple), and PID-HIF2 PATHWAY (blue) in the PBRM1^MUT^ ccRCC tumor samples (Figure 3F). FARDIN hypoxia signaling, MENSE hypoxia signaling, MIZUKAMI hypoxia signaling, PID-HIF1-THPATHWAY, and PID-HIF2 PATHWAY pathways were all predicted activation of hypoxia- and HIF-related signaling pathways in the PBRM1^mut^ group of ccRCC patients. We further analyzed the expression of angiogenesis-related genes, such as, VEGFA, VEGFB, VEGFC, VCAM1 (vascular cell adhesion molecule 1), PDGFA (platelet derived growth factor A), and PDGFB (platelet derived growth factor B) in the GSE36895 and TCGA ccRCC patient cohorts. As shown in Table 1, *PBRM1*^MUT^ patients in both cohorts showed significantly higher VEGFA, VEGFB, VEGFC, VCAM1, PDGFA, and PDGFB mRNA expression compared to the *PBRM1*^WT^ patients. These results suggest that *PBRM1* mutation promotes mast cell infiltration into the TME and activates HIF-related signaling pathways that drive growth and progression of ccRCC.

**Table 1:** Angiogenesis-related genes were upregulated in PBRM1^MUT^ patients in 2 cohorts.

	**GSE36895**		**TCGA**	
	**PBRM1^MUT^ (13)/PBRM1^WT^ (16)**		**PBRM1^MUT^ (131)/PBRM1^WT^ (177)**	
	**Fold change**	**p value**	**Fold change**	**p value**
VEGFA	1.27	0.14	1.14	0.04
VEGFB	1.21	0.23	1.12	0.01
VEGFC	1.12	0.65	1.01	0.84
VCAM1	2.00	0.03	1.00	1.00
PDGFA	1.36	0.06	1.10	0.07
PDGFB	1.01	0.80	1.10	0.08

### Low PBRM1 protein expression is associated with increased infiltration of mast cells

**Figure 4:**
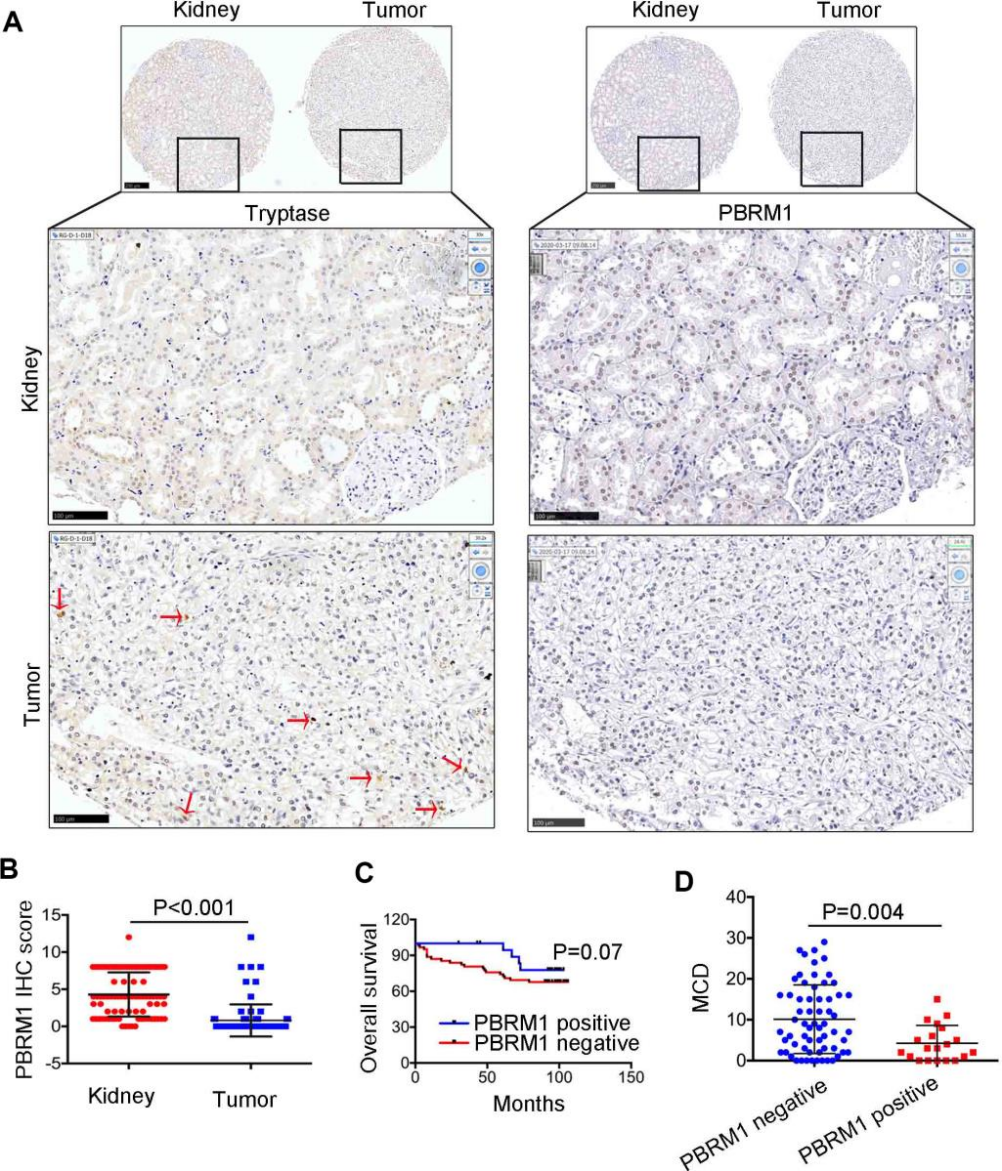
**The relationship between PBRM1 protein expression and mast cell infiltration in ccRCC based on IHC analysis.** (**A**) Representative immunohistochemical images show PBRM1- and tryptase-positive mast cells in ccRCC and adjacent normal kidney tissue samples. (**B**) Dot plot of PBRM1 IHC staining score in adjacent normal kidney tissues (n=83) and ccRCC tissues (n=83). (**C**) Overall survival of ccRCC patients with PBRM1 IHC staining negative group (n=65) or PBRM1 IHC staining positive group (n=20). (**D**) Pearson correlation analysis shows the association between PBRM1 expression and mast cell infiltration in 85 out of 90 ccRCC patient tumor tissue samples. Data for five tumor tissues is not included (missing the tissues in TMA). Note: Statistical significance was based on Student’s t-test.

**Figure 5:**
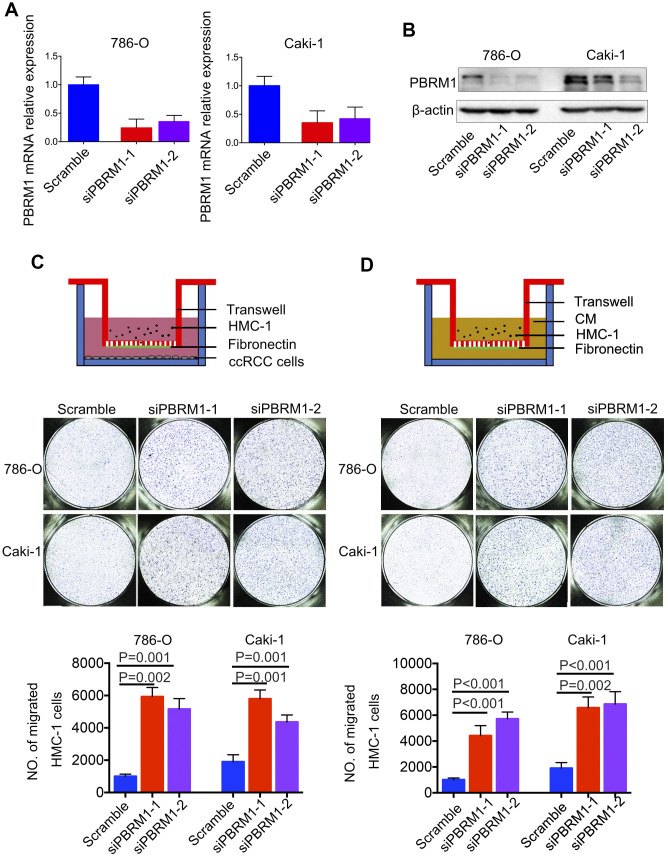
**PBRM1-silenced ccRCC cells recruit significantly higher numbers of mast cells *in vitro*.** (**A**) qRT-PCR and (**B**) Western blot analysis shows PBRM1 mRNA and protein levels in control and PBRM1-silenced 786-O and Caki-1 cells. (**C**, **D**) Transwell migration assay results show the total numbers of migrating HMC-1 cells when co-cultured with control and PBRM1-silenced 786-O and Caki-1 cells or the conditioned media from these cells. The migrating HMC-1 cells are stained with crystal violet and counted. The experiments were performed in triplicate and the results are shown as means±SD. Student’s t-test was used to determine statistical significance.

Next, we analyzed the association between PBRM1 protein levels and mast cell infiltration in ccRCC tumor samples. Immunohistochemical analysis (IHC) of 90 ccRCC and adjacent normal kidney tissues using anti-PBRM1 and anti-Tryptase (specific marker of mast cells) antibodies showed that PBRM1 protein expression was significantly reduced in the ccRCC tissues compared to the adjacent kidney tissues (Figure 4A, 4B, P<0.001). Moreover, as shown in Figure 4A, normal kidney tissues were PBRM1-positive and Tryptase-negative (no mast cells in normal tissue), whereas, ccRCC tumor tissues were PBRM1-negative and Tryptase-positive (high numbers of mast cells in the tumor). Furthermore, 65 out of 85 (76.4%) ccRCC patients that were PBRM1-negative showed worse overall survival than the remaining PBRM1-positive ccRCC patients (Figure 4C), however, there was no statistically significant between two groups (P=0.07). Moreover, tumor tissues from PBRM1-negative ccRCC patients showed significantly higher mast cell infiltration compared to those from PBRM1-positive ccRCC patients (Figure 4A, 4D). To confirm the effects of PBRM1 on mast cell recruitment, we performed an *in vitro* Transwell migration assays using 5×10^4^ HMC-1 cells in the upper chamber and PBRM1-knockdown ccRCC cells (786-O and Caki-1, Figure 5A, 4B) or conditioned media from these cells in the lower chamber. The results of the co-culture Transwell migration assay showed that HMC-1 recruitment was significantly higher in PBRM1-silenced 786-O- (siPBRM1-1, P=0.003; siPBRM1-2, P=0.006) and PBRM1-silenced Caki-1 (siPBRM1-1, P=0.002; siPBRM1-2, P=0.004) cells compared to their corresponding siNC-transfected controls (Figure 5C). Furthermore, HMC-1 recruitment was significantly higher in conditioned media derived from the PBRM1-silenced 786-O (siPBRM1-1, P=0.007; siPBRM1-2, P=0.005) and PBRM1-silenced Caki-1 (siPBRM1-1, P=0.001; siPBRM1-2, P=0.001) cells compared to CM derived from siNC-transfected controls (Figure 5D). These data confirm that PBRM1 downregulation in ccRCC cells promotes recruitment of mast cells, both *in vivo* and *in vitro*.

### PBRM1 silencing enhances tumor angiogenesis and ccRCC cell proliferation *in vitro*

**Figure 6:**
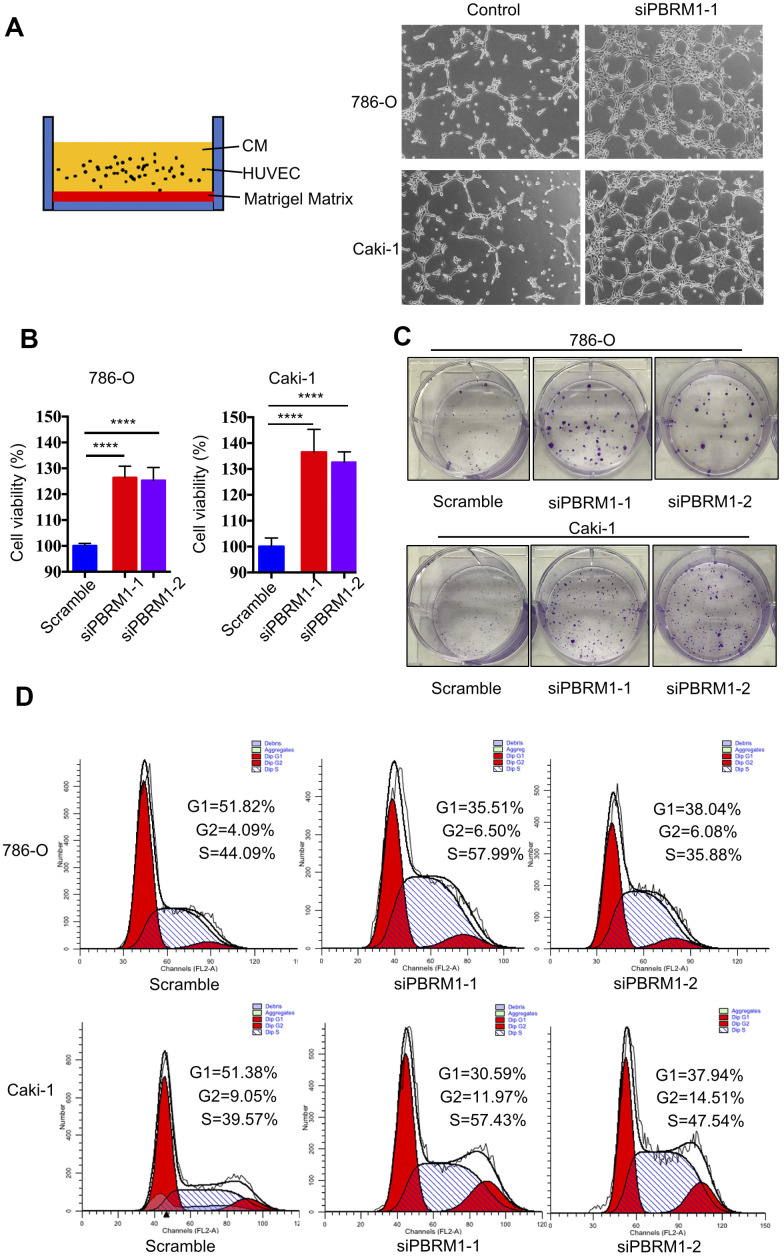
**PBRM1 silencing enhances tumor angiogenesis and promotes cell proliferation of RCC cells in vitro.** (**A**) Matrigel tube formation assay results show the tube-like structures formed in the matrigel by CM from control and PBRM1-silenced 786-O and Caki-1 cells. (**B**) MTT assay results show viability of control and PBRM1-silenced 786-O and Caki-1 cells. (**C**) The colony formation assay results show the total numbers of colonies formed by control and PBRM1-silenced 786-O and Caki-1 cells based on crystal violet staining. (**D**) Flow cytometry analysis shows the percentage of G1, S, and G2-M cells in control and PBRM1-silenced 786-O and Caki-1 cells based on PI staining. Note: The experiments were performed in triplicate and data are represented as means±SD; the statistical analysis was performed using Student’s t-test.

Next, we performed tube formation matrigel assay to determine the effects of PBRM1-knockdown in ccRCC cells on tumor angiogenesis. The tube formation assay results showed that PBRM1-silenced 786-O (P=0.0067) and PBRM1-silenced Caki-1 (P=0.0034) cells formed higher numbers of tube-like structures compared to the corresponding controls (Figure 6A and Supplementary Figure 4A).

We also verified the results of PBRM1 suppression ccRCC cell growth in Varela et al*.* report [13]. The cell growth rate and colony formation ability was significantly higher in the PBRM1-knockdown 786-O and Caki-1 cells compared to the corresponding controls (Figure 6B, 6C and Supplementary Figure 4B). Furthermore, the proportion of G1-phase cells were significantly reduced in the PBRM1-knockdown 786-O and Caki-1 cells compared to the siNC-transfected 786-O and Caki-1 cells (786-O: 51.82% for control vs. 35.51% for siPBRM1-1 and 38.04% for siPBRM1-2; Caki-1: 51.38% for control vs. 30.59% for siPBRM1-1 and 37.94% for siPBRM1-2; Figure 6D). This suggests that PBRM1 silencing enhances G1-S transition of ccRCC cells, thereby increasing cell proliferation. Overall, these results demonstrate that PBRM1 silencing enhances angiogenesis and ccRCC cell proliferation.

### PBRM1 silencing promotes mast cell recruitment involving upregulated CCL5 in the ccRCC tumor microenvironment

**Figure 7:**
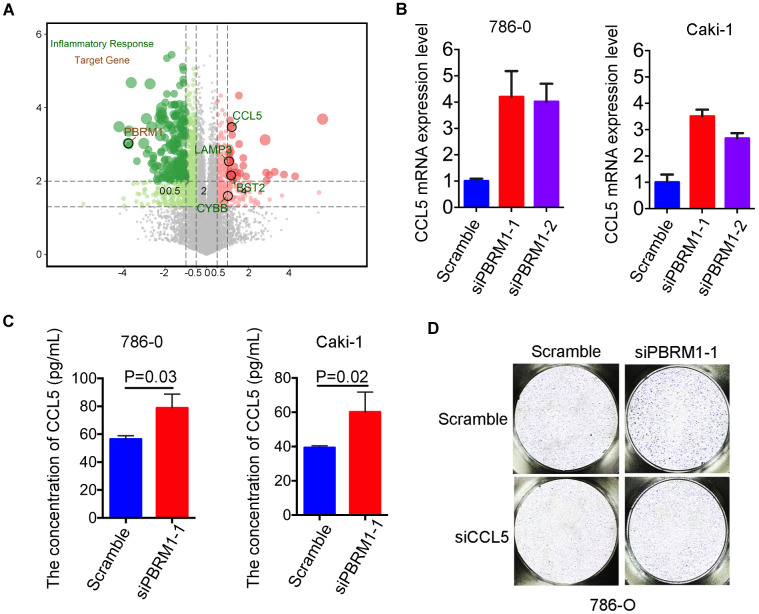
**High CCL5 expression and secretion correlates with mast cell infiltration in *PBRM1*^MUT^ccRCC cells and patients.** (**A**) Volcano plot shows fold changes in gene expression in control and PBRM1-overexpressing Caki-2 cells. The association of immune response with mutations in *PBRM1*, *VHL*, *SETD2* and *BAP1* genes is shown in black circles. (**B**) qRT-PCR analysis shows CCL5 mRNA expression in 786-O- and Caki-1-silenced PBRM1 cells. (**C**) ELISA assay results show CCL5 levels in the conditioned media of control and PBRM1-silenced 786-O and Caki-1 cells using the human CCL5 ELISA kit. (**D**) Transwell migration assay results show total numbers of migrating HMC-1 cells when co-cultured with conditioned media derived from control, PBRM1-silenced and PBRM1-silenced plus CCL5-silenced 786-O cells. The migrating MHC-1 cells were stained with crystal violet and counted. Note: All experiments were performed in triplicate and are presented as means±SD; statistical analysis was performed using Student’s t-test.

We next sought to understand the molecular mechanisms through which PBRM1-silenced ccRCC cells promote infiltration of mast cells. Pathway analysis showed that the inflammatory response signaling pathway was suppressed in PBRM1-overexpression Caki-2 cells (P<0.001, GSE76199; Supplementary Figure 5). Moreover, CCL5, the mast cell chemoattractant cytokine, was down-regulated in PBRM1-overexpression Caki-2 cells, and also CCL5 is one of the genes listed in the inflammatory response pathway (Figure 7A). qRT-PCR analysis showed that CCL5 mRNA levels were significantly upregulated in PBRM1-silenced 786-O and Caki-1 cells compared to the corresponding controls (Figure 7B). Moreover, ELISA assay results showed that CCL5 protein levels were significantly higher in the conditioned media of PBRM1-silenced ccRCC cells compared to the conditioned media of the controls (786-O: P=0.04; Caki-1: P=0.02; Figure 7C).

In order to further examine the biological function of CCL5 on mast cell recruitment in renal carcinoma, CCL5 was knocked down in 786-O and Caki-1 (Supplementary Figure 4C). Transwell migration assay showed that the numbers of migratory HMC-1 cells were decreased when co-cultured with conditioned media of CCL5-silenced cells compared to the conditioned media of control cells in 786-O and Caki-1 cells, respectively (Supplementary Figure 4D, 4E). Furthermore, we constructed CCL5 knockdown in PBRM1-silenced ccRCC cells. As we expected, the numbers of migratory HMC-1 cells were significantly higher when co-cultured with conditioned media of PBRM1-silenced 786-O cells compared to the conditioned media of control 786-O cells (Figure 7D, Supplementary Figure 4F, P=0.002). Furthermore, CCL5 knockdown in PBRM1-silenced 786-O cells reversed the migration of HMC-1 cells through the transwell membrane compared to PBRM1-silenced 786-O cells (Figure 7D and Supplementary Figure 4F). CCL5 knockdown inhibited the numbers of migratory HMC-1 cells were also verified in PBRM1-silenced Caki-1 cells (Supplementary Figure 4G, 4H). These data show that PBRM1-silenced ccRCC cells produce significantly higher levels of CCL5, and CCL5 was involved in promoting recruitment of mast cells.

### High CCL5 expression is associated with adverse survival outcomes in ccRCC patients

**Figure 8:**
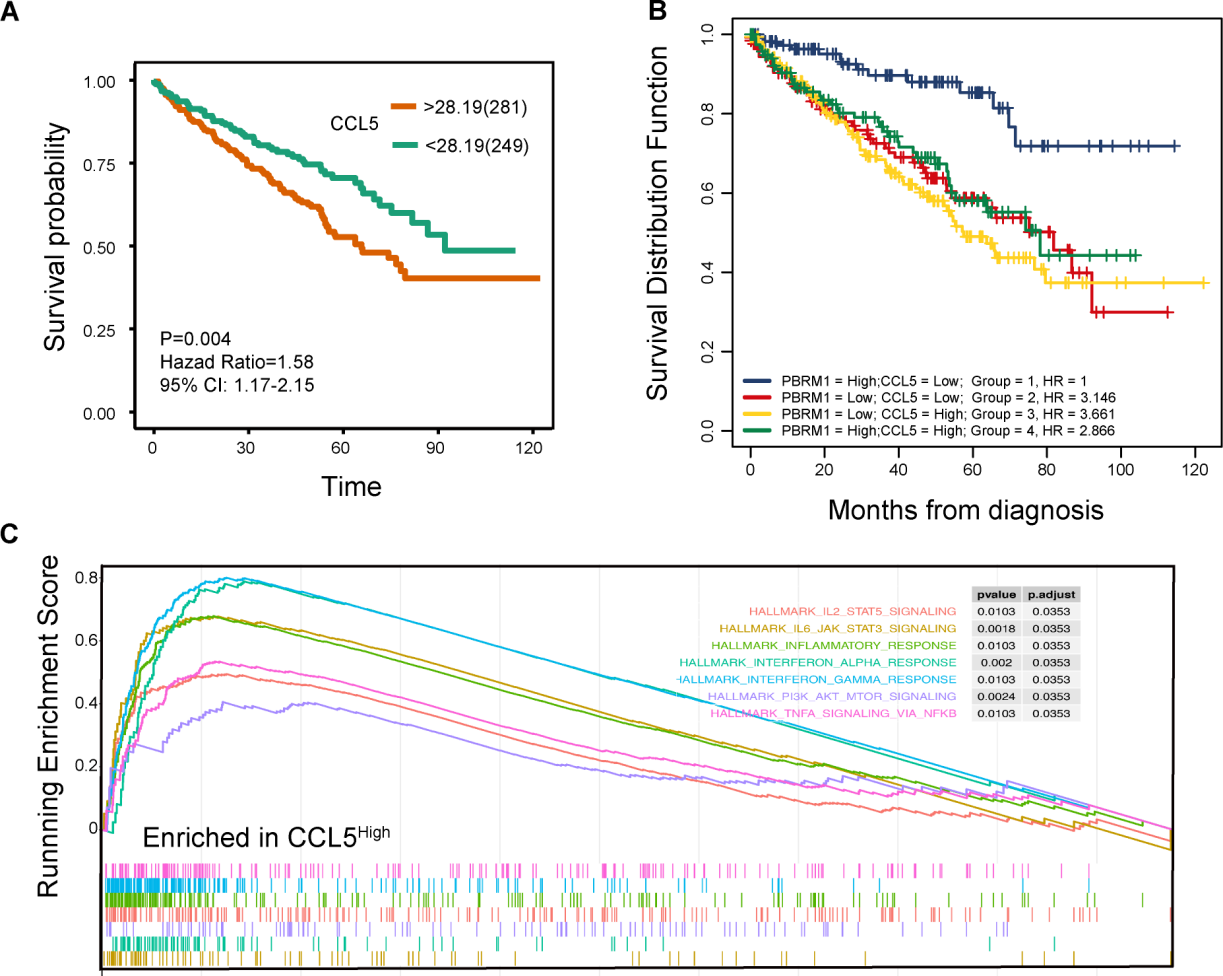
**High CCL5 expression is associated with immune suppression and adverse survival outcomes in ccRCC.** (**A**) The overall survival (OS) of high- and low-CCL5 expressing ccRCC patients in the TCGA KIRC database as evaluated by the survival and survminer packages is shown. P<0.05 is considered statistically significant. (**B**) The overall survival of ccRCC patients in the TCGA KIRC database according to high- and low- PBRM1 and CCL5 expression using survminer packages, log-rank tests, and COX regression analysis. (**C**) Enrichment plots show the status of gene sets belonging to IL6/JAK/STAT3 signaling (yellow), IL2/STAT5 (red), the inflammatory response (green), the IFN-α response (light blue), the IFN-γ response (blue), PI3K/AKT/MTOR signaling (purple), and TNF-α/NFΚB signaling (light red) pathways in the CCL5^High^ group of ccRCC patients.

Next, we analyzed the relationship between CCL5 expression and ccRCC prognosis. Survival analysis showed that ccRCC patients with high CCL5 expression (CCL5^High^) were associated with worse survival outcomes than those with low CCL5 expression (CCL5^Low^; Figure 8A; P=0.004). Moreover, PBRM1^High^/CCL5^Low^ ccRCC patients exhibited better overall survival than PBRM1^High^/CCL5^High^, PBRM1^Low^/CCL5^High^, and PBRM1^Low^/CCL5^High^ ccRCC patients (Figure 8B). Furthermore, the hazard ratios (HR) of ccRCC patients with PBRM1^High^/CCL5^High^, PBRM1^Low^/CCL5^High^, and PBRM1^Low^/CCL5^High^ were 2.866 (P<0.001), 3.661 (P<0.0001), and 3.146 (P<0.001) higher than the HR of ccRCC patients with PBRM1^High^/CCL5^Low^ expression (Figure 8B). As shown in Figure 8C, CCL5^high^ ccRCC patients showed significant enrichment in immune-related signaling pathways, including inflammatory signaling (P=0.0103), IFN-γ signaling (P=0.0103), IFN-α signaling (P=0.002), IL-6-JAK-STAT3 (P=0.0018), and TNF-α-NF-ΚB signaling (P=0.0103).

We also evaluated the clinical significance of IFN-γ expression in ccRCC patients. Survival analysis showed that low IFN-γ-expressing ccRCC patients (IFN-γ^Low^) showed better outcomes than IFN-γ^High^ ccRCC patients (P=0.004; Supplementary Figure 6A). Moreover, CCL5^Low^/IFN-γ^Low^ ccRCC patients showed better overall survival than CCL5^High^/IFN-γ^Low^, CCL5^Low^/IFN- γ^High^, and CCL5^High^/IFN-γ^High^ ccRCC patients (Supplementary Figure 6B). Furthermore, PBRM1^High^/IFN-γ^Low^ ccRCC patients showed better overall survival than PBRM1^Low^/IFN-γ^Low^ PBRM1^High^/IFN-γ^High^ and PBRM1^Low^/IFN-γ^High^ ccRCC patients (Supplementary Figure 6C). Overall, our results confirm that high CCL5 expression promotes immune suppression and is positively associated with adverse outcomes in ccRCC patients.

## DISCUSSION

Patients with metastatic ccRCC are associated with poor prognosis because of a high frequency of drug resistance. Hence, several studies are investigating the mechanisms that regulate ccRCC progression in order to identify new therapeutic targets [40–42]. Drugs that target aberrantly upregulated VEGF and mTOR signaling pathways because of VHL mutations show limited antitumor activity in ccRCC patients [6]. Previous studies show that PBRM1 modulates ccRCC progression and immunotherapy response [25, 43], but, the mechanisms are not clear. In this study, we demonstrate that PBRM1^MUT^ ccRCC tumors overexpress VEGF and related proteins and activate HIF-related signaling pathways. Furthermore, PBRM1^MUT^ ccRCC tumors recruit significantly higher numbers of mast cells into the tumor microenvironment, and CCL5 was upregulated in siPBRM1 cells, suggesting that mast cells were recruited by CCL5.

We also demonstrate that the infiltration of 22 different types of immune cells into the tumor microenvironment is dependent on the status of *PBRM1* mutations (Figure 2B, 2D) and independent of *VHL* mutations (Figure 2A). This is consistent with the findings of Miao et al*.* who reported that the expression of immune-related factors such as IFNγ, CD8α, CD47, and IL10 are associated with *PBRM1* mutations, but independent of *VHL* mutation status [25]. Deng et al*.* showed that *PBRM1* deficiency increases the numbers of M2-like macrophages and dendritic cells in the tumor micro-environment [24]. Together, these results demonstrate that *PBRM1* promotes ccRCC progression by modulating immune cell infiltration.

Analysis of the ccRCC TCGA datasets demonstrate that the numbers of CD8^+^T cells are significantly reduced in PBRM1^MUT^ ccRCC patients (Figure 2B). Moreover, PBRM1^MUT^ ccRCC tumors are associated with lower immunoscores (P=0.001) and ESTIMATES scores (P=0.007; Figure 2C), indicated *PBRM1* mutation predicted worse response to PD-1 blockade. However, a study by Miao et al. showed that PBRM1 deficiency is associated with intermediate (n=17) or significant (n=27) response to anti-PD-L1 therapy in a cohort of 63 ccRCC patients, thereby contradicting with our findings [25]. Moreover, 25 of these 44 (57%) patients had an intact *PBRM1* gene, with nonsense mutations in 5 patients. Miao’s study was consistent with David’s report, which identified that CD8^+^T cell infiltrated tumors are relatively depleted for *PBRM1* mutations, and CD8^+^T cell infiltration by itself is not associated with response to anti-PD-1 [27]. Taken together, these contradictory results suggest that further multicenter large cohort studies are required to assess the relationship between *PBRM1* mutation status and clinical benefit from immune therapy in ccRCC patients.

The study by Miao et al. also showed that IL-6-JAK-STAT3, TNF-α, and IFN-γ pathways were activated in PBRM1 deficient patients; upregulation of the IFN-γ signaling pathway increased the surface expression of PD-L1, which was the target of anti-PD-L1 therapy [25]. Our study also shows that the immune-related interferon, IFN-γ, and IL-6-JAK-STAT3 signaling pathways were all activated in PBRM1^MUT^ ccRCC tumor tissues (Figure 8C). However, in David’s study, they found PBRM1 alterations were associated with lower IL6–JAK–STAT3 signaling [27].

In our study, we performed IHC as a ‘gold standard’ approach to test the relationship between PD-L1 expression and PBRM1 expression. 90 ccRCC tumor samples in a tissue array and found no correlation between PBRM1 and PD-L1 protein expressions (Supplementary Figure 7A). There were no significant differences in PD-L1 expression between PBRM1-negative and PBRM1-positive samples in the 90 ccRCC tissue array samples (Supplementary Figure 7B). PD-L1 expression was also not upregulated in the PBRM1^MUT^ ccRCC patients from the TCGA dataset (Supplementary Figure 7C). Motzer et al. showed that PD-L1 was not a good biomarker for immunotherapy in ccRCC patients based on IHC analysis [9]. Chen et al*.* reported that IFN-γ upregulates PD-L1 on exosomal vesicles, which inhibits the function of CD8^+^T cells, thereby facilitating melanoma progression [44]. We also showed that treatment of ccRCC cells with 10 ng/μL IFN-γ increased the expression of exosomal PD-L1 (Supplementary Figure 7D, 7E). This suggests that elevated IFN-γ signaling in ccRCC cells upregulates exosomal PD-L1 expression and inhibits CD8^+^T cells in the TME.

Our study showed that infiltration of resting mast cells was significantly enriched in PBRM1^MUT^ccRCC patients (Figures 1B, 2B, 2D). This was further confirmed by *in vitro* Transwell migration experiments that showed significant increase in HMC-1 cell migration when co-cultured with CM from PBRM1-silenced ccRCC cells (Figure 5C, 5D). Moreover, PBRM1 down expression correlated with increased CCL5 expression (Figure 7A, 7B). But, there was no change in SCF levels in PBRM1-silenced ccRCC cells and in PBRM1^MUT^ccRCC patients from the TCGA dataset. CCL5 is a chemoattractant for mast cells, which function as immunosuppressive cells that inhibit natural killer (NK) cells and other immune cells [31, 32]. We demonstrate that NK cells are inhibited in PBRM1^MUT^ccRCC patients (Figure 2B). Mast cells secrete TNF-α, an inducer for tumor angiogenesis [31]. Our study demonstrates that TNF-α signaling pathway is activated in PBRM1^MUT^ ccRCC patients (Figure 8C), and also consisted with our tube formation results (Figure 6C). Mast cells also suppress T cells, which are a critical subset of cells that suppress tumor growth and progression [31]. Our results demonstrate reduced numbers of CD8^+^T cells and increased numbers of mast cells in PBRM1^MUT^ccRCC patients (Figure 2B). Our results are consistent with the study by Xiong et al*.*, which reports that mast cells reduce CD8^+^T cell infiltration, and aberrant production of immunosuppressive cytokines, IL-10 and TGF-β in the ccRCC TCGA KIRC and SATO cohorts [36]. Mast cells promote tumor microvessel density by activating the PI3K-AKT-GSK3-AM signaling pathway [37]. They also promote metastatic escape of tumor cells by upregulating matrix metalloproteases like MMP9 [31, 45]. They are also involved in the regulation of extracellular matrix remodeling, which is necessary for tumor metastasis [36]. However, the interaction of mast cells with other cell types in the tumor microenvironment remains unclear, and is an active area of research because its understanding may benefit cancer prognosis and therapy.

**Figure 9:**
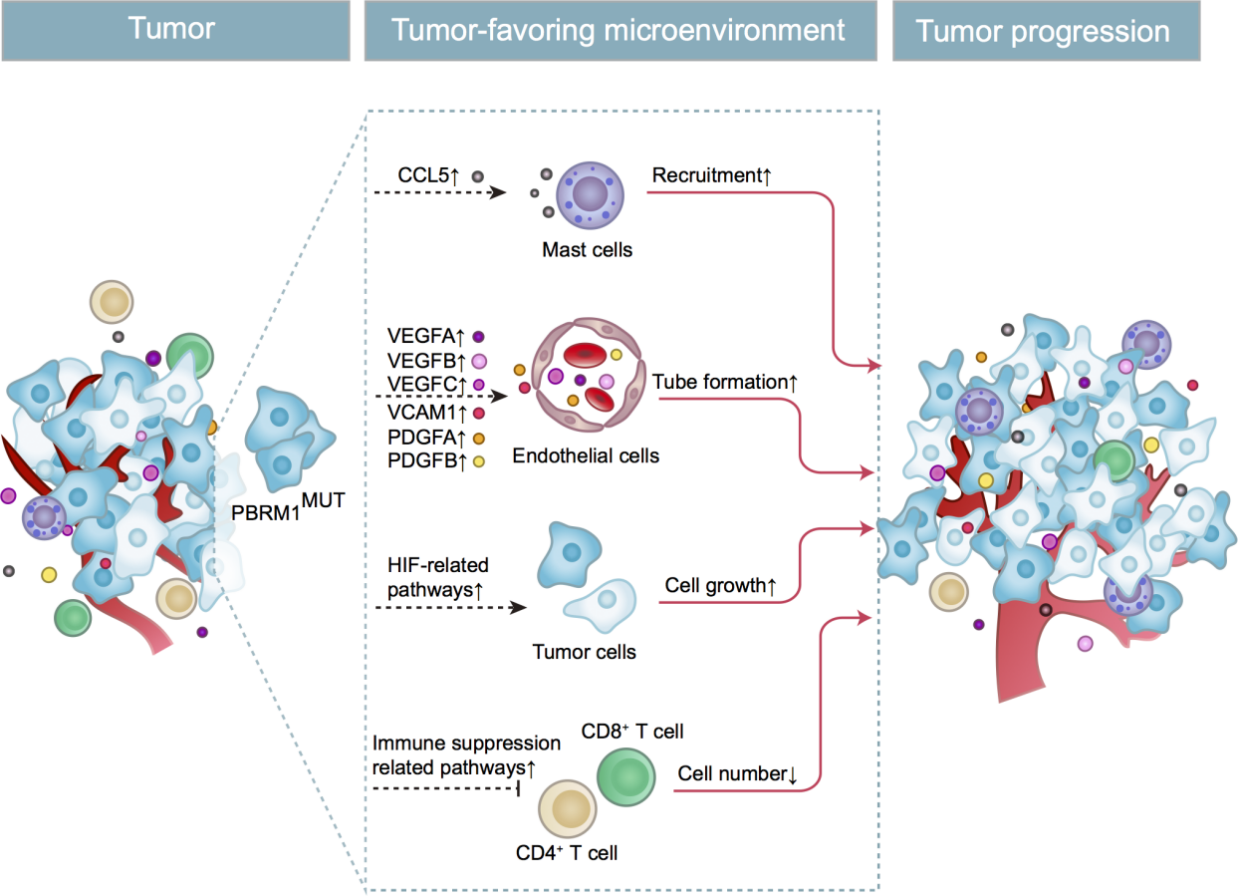
**Schematic representation shows the mechanism through which *PBRM1*^MUT^ ccRCC cells modulate tumor microenvironment and promote ccRCC progression.** The PBRM1 mutant ccRCC cells secrete CCL5 cytokines that promote mast cell recruitment into the TME. The mast cells secrete several factors such as VEGFA, VCAM1, and PDGFA that stimulate angiogenesis. The mast cells also reduce the infiltration of CD8^+^ T cells and CD4^+^ T cells. Simultaneously, *PBRM1* mutations facilitate tumor cell growth by activating intrinsic HIF signaling pathways. The complex interactions between the mast cells, epithelial cells, T cells, and ccRCC tumor cells in the TME are aided by several cytokines and chemokines that are secreted by these cells regulates tumor progression.

In conclusion, as shown in Figure 9, we demonstrate that *PBRM1*^MUT^ ccRCC patients produce and secrete excessive CCL5, which recruits higher number of mast cells into the tumor microenvironment. The mast cells secrete excessive immunosuppressive cytokines like IL-10 and TGF-β, which inhibits CD8^+^ T cell infiltration and function. This contributes to increased growth and progression of *PBRM1*^MUT^ ccRCC.

## MATERIALS AND METHODS

### Patient datasets and preliminary

We downloaded the transcriptome (count and FPKM value), clinical, and mutational data for 539 ccRCC patients from The Cancer Genome Atlas (TCGA, https://www.cancer.gov/tcga) database and for the GSE76199 and GSE36895 datasets from the gene expression omnibus (GEO: https://www.ncbi.nlm.nih.gov/geo/) database [46, 47]. The wild-type *PBRM1* (PBRM1^WT^) and mutant *PBRM1* (PBRM1^MUT^) patient groups were determined by first removing the low-value genes using heterogeneity analysis followed by normalizing the data sets using the variance stabilizing transformation (VST) method in the DESeq2 package as previously described [48]. Finally, the two groups of data were hierarchically clustered, and the heterogeneous samples in the two groups were excluded. The wild-type *VHL* (VHL^WT^) and mutant *VHL* (VHL^MUT^) patient groups were established as described above for *PBRM1*. The GSE76199 dataset also contained transcriptome data for PBRM1-overexpressing Caki-2 cell lines.

### Identifying top 20 gene mutations in the TCGA ccRCC dataset

Maftools was used to identify the top 20 gene mutations in the ccRCC dataset of the TCGA KIRC (kidney renal clear cell carcinoma) database [49].

### Analysis of tumor infiltration of immune cells

To determine infiltration of twenty-two types of immune cells in ccRCC, we converted the count values in the TCGA KIRC transcript data into TPM (transcripts per kilobase of exon model per million mapped reads) values. Then, we removed outlier samples and calculated the infiltration of the 22 immune cells in 178 samples including those with wild-type and mutant PBRM1 with the CIBERSORT algorithm (https://cibersort.stanford.edu/) using 1000 permutations and the LM22 expression matrix [50]. The immune cell fractions were considered accurate when the CIBERSORT output reached p < 0.05.

### Immune and stromal purity score analysis using ESTIMATE algorithm

The transcriptome data was analyzed using ESTIMATE algorithm to determine stromal and immune purity scores for the ccRCC samples from the TCGA KIRC database [39]. The stromal and immune purity scores for the PBRM1^WT^ and PBRM1^MUT^ groups were expressed as mean ± standard error (S.E).

### Gene clusters and coexpression network analysis

We analyzed gene expression data of 178 ccRCC samples with the Weighted Correlation Network Analysis (WGCNA) package and identified 28 gene cluster modules using softpower = 6 [51]. Then we established co-expression networks within these 28 gene cluster modules to determine the correlation between immune cell infiltration and gene mutations (*VHL*, *PBRM1*, *SETD2*, and *BAP1*).

### Identification of differentially expressed genes

The Robust Multiarray Average (RMA) was used to normalize the GSE36895 chip data, and KNN (k-NearestNeighbor) method was performed to calculate and complement the values. Then, differentially expressed genes (DEGs) in the PBRM1^WT^ and PBRM1^MUT^ groups from the GSE36895 dataset were identified using the limma package with P<0.05, adjusted P<0.05, and |fold change|>2 as threshold parameters [52]. For the TCGA transcriptome dataset, edgeR package was used to identify the DEGs between the PBRM1^WT^ and PBRM1^MUT^ groups with P<0.05, FDR<0.05, and |fold change|>2 as threshold parameters [53].

### Functional enrichment analysis

Metascape was used for data visualization of functional enrichment analysis [54]. Kyoto Encyclopedia of Genes and Genomes (KEGG) analysis was used to analyze the DEGs and determine the reactome, hallmark, and canonical gene sets based on threshold parameters, including P<0.01, minimum overlap>3, and minimum enrichment>1.5. Furthermore, gene set enrichment analysis (GSEA) was performed using edgeR and clusterProfiler packages to determine significantly altered pathways between the PBRM1^WT^ and PBRM1^MUT^ groups and the CCL5^High^ and CCL5^Low^ groups in the ccRCC samples of the TCGA dataset [55, 56].

### Survival analysis

The survival and survminer packages were used to perform 2-gene survival analysis and determine the effects of CCL5, PBRM1, and IFN-γ (interferon-gamma) on the overall survival of ccRCC patients from the the TCGA dataset. The survival curves were analyzed using the log-rank test. COX regression analysis was used to calculate the corresponding HR value in the PBRM1/CCL5 and CCL5/IFN-γ 2-gene survival analysis. The HR value of the control group (PBRM1^High^/CCL5^Low^, CCL5^High^/ IFN-γ^Low^, PBRM1^High^/IFN-γ^Low^, respectively) was 1.

### Cell lines and cell culture

We purchased the human renal cancer cell lines, Caki-1 and 786-O, human mast cell line, HMC-1, and human umbilical vein endothelial cell (HUVEC) from the American Type Culture Collection (ATCC, Manassas, VA, USA). The cell lines were grown in RPMI 1640 or IMDM medium (Gibco-Thermo Fisher Scientific, Inc., Waltham, MA, USA), supplemented with 10% (v/v) fetal bovine serum (FBS; Gibco-Thermo Fisher Scientific, Inc., Waltham, MA, USA) at 37°C and 5% CO_2_ in a humidified incubator.

### Transfections

The ccRCC cells were cultured in 6-well culture dishes for 24 h and transfected with the siRNA against PBRM1, or siCCL5 (si-PBRM1 and siCCL5; Ribobio, Guangzhou, China) using X-tremeGENE siRNA transfection reagent kit (Roche Co. Ltd., Shanghai, China) according to the manufacturer’s protocol. The transfection efficiency was verified using quantitative real-time PCR (qRT-PCR) and western blotting analyses.

### Real-time quantitative PCR

Total cellular RNA was isolated using the RNA Fast 200 kit (Feijie Biotech, Shanghai, China) according to manufacturer’s protocol. The cDNA synthesis was performed using Prime Script™ RT reagent kit (Takara Biotechnology Co. Ltd., Dalian, China) and subsequently, qPCR was performed using the SYBR Green PCR Master Mix (Takara Biotechnology Co. Ltd., Dalian, China). The relative expression of PBRM1 and CCL5 was calculated by the 2^–ΔΔCt^ method [57]. GAPDH was used as the internal control. The primers used for qPCR are as follows: GAPDH forward: 5’-AT GGGGAAGGTGAAGGTCGG-3’; GAPDH reverse: 5’-GACGGTGCCATGGAAT TTGC-3’; PBRM1 forward: 5’-AGGAGGAGACTTTCCAATCTTCC-3’; PBRM1 reverse: 5’-CTTCGCTTTGGTGCCCTAATG-3’; CCL5 forward: 5’-CCAGCAGTCGTCTTTGTCAC-3’; CCL5 reverse: 5’-CTCTGGGTTGGCACACACTT-3’.

### MTT assay

We performed 3-(4,5-dimethyl-2-thiazolyl)-2,5-diphenyl-2-H-tetrazolium bromide (MTT) cell viability assay as previously described [58]. Briefly, we seeded 4×10^3^ si-PBRM1 and si-NC-transfected cells in a 96-well plate for 48 h. Then, we added 10 μl of MTT to each well and incubated for 4 h. The OD was measured at 450 nm in a plate reader. The cell viability rate was calculated as the average OD value of the siPBRM1 group/average OD value of the siNC group × 100%.

### Western blotting analysis

Western blotting was performed as described previously [59]. Briefly, total protein lysates were prepared using RIPA buffer and quantified using BCA protein assay (#23225, Thermo Scientific Inc., IL, USA). Then, equal amounts of proteins were separated on 12% SDS-PAGE followed by transfer onto nitrocellulose membranes. Then, the membranes were blocked with 5% skimmed milk for 1 h at room temperature. Then, the membranes were incubated overnight with primary rabbit anti-human PBRM1 antibody (#A10009; 1:1000; ABclonal, Wuhan, China) and anti-β-actin antibody (#JB09, 1:1000, Absin, Shanghai, China) as an internal control. Later, the membranes were incubated with horseradish peroxidase (HRP)-conjugated goat anti-rabbit IgG (#ZB-2301, 1:2,000, Beijing Zhongshan Golden Bridge Biotechnology, Co. Ltd., Beijing, China) or HRP-conjugated goat anti-mouse IgG (#ZB-2305, 1:2000, Beijing Zhong-shan Golden Bridge Biotechnology, Co. Ltd., Beijing, China) for 1 h at room temperature. The blots were developed using the Supersignal ^TM^ West Pico Plus Chemiluminescent Substrate kit (#34580, Thermo Scientific Inc., IL, USA) according to manufacturer’s instructions and the protein bands were visualized using the Molecular Imager ChemiDoc XRS system (Bio-Rad Laboratories, Inc., Hercules, CA, USA). The PBRM1 protein levels were quantified relative to β-actin levels in each sample using the Image J software.

### Conditioned media

We seeded 40×10^4^ control and PBRM1 knockdown cells in 6-cm culture dishes for 24 h. Then, the cells were washed twice with serum-free medium (SFM) and further incubated for 24 h with 3 mL SFM. Then, we centrifuged the conditioned media to remove all cell debris and stored the CMs at −80°C for further experiments.

### Colony formation assay

We cultured 1× 10^3^ control and PBRM1 knockdown cells in 6-well plates for 7 days at 37^o^C and 5% CO_2_. Then, the colonies were stained with 0.5% w/v crystal violet and under a light microscope (Nikon ECLIPSE Ts2R, Japan).

### Cell cycle analysis

The si-NC- and si-PBRM1-transfected ccRCC cells were washed with phosphate-buffered saline (PBS) and permeabilized with pre-chilled 70% ethanol at -20°C overnight. The cells were then washed twice with PBS and incubated with propidium iodide (PI) in the dark for 30 min as previously described [59]. Then, the cell cycle distribution of samples was analyzed using a FACSCalibur flow cytometer (BD Biosciences, San Jose, CA) and the CELLFIT software was used to examine the cell cycle distribution.

### Mast cell recruitment assay

We used a 24-well transwell co-culture assay to estimate *in vitro* mast cell recruitment. Briefly, 10× 10^4^ cells PBRM1-knockdown ccRCC cells (786-O and Caki-1) were seeded into the lower chamber, after 24 h, the bottom of a 8 μm polycarbonate filter was coated with 10 μg/ml fibronectin (sc-29011 Santa Cruz Biotechnology) at 37°C and 5% CO_2_ in a humidified incubator for 4 h, 5×10^4^ HMC-1 cells were seeded in the upper chambers. For conditioned media recruitment, the lower chamber was added 900 μL of pre-warmed conditioned media from PBRM1-knockdown or control ccRCC, and incubated at 37°C and 5% CO_2_ in a humidified incubator for 24 h. Then, the migrating HMC-1 cells at the bottom side of the filters were fixed with 4% paraformaldehyde for 10 mins, washed thrice with PBS, and stained with 0.5% w/v crystal violet for 15 mins. Then the total numbers of migrating HMC-1 cells were measured in 3 random fields to estimate mast cell recruitment efficiency.

### ELISA assay

We used the Proteintech human CCL5 ELISA Kit (#KE00093, Proteintech Inc., IL, USA) to measure human CCL5 levels in the conditioned media from control and PBRM1 knockdown ccRCC cells according the manufacturer’s instructions.

### Tube formation assay

A 24-well plant was coated with matrigel (BD Biosciences, Franklin Lakes, NJ, USA) for 4 h at 37 °C, then HUVECs (1×10^5^/well) were suspended in CMs and seeded into 24 well plate. After 3 h, photographs were taken under a light microscope (Nikon ECLIPSE Ts2R, Japan).

### Immunohistochemistry

We purchased tissue microarrays (#HKidE180Su03, including 90 adjacent kidney tissues and 90 ccRCC patients) from Shanghai Outdo BioTeck Co. Ltd (Shanghai, China) and performed immunohistochemical staining of PBRM1 (Abcam, dilution 1:200, #ab196022) and Tryptase (Abcam, dilution 1:500, #ab134932) using a DAKO Autostainer Plus system (#GK600505, Gene Tech Company, Shanghai, China). Immunohistochemical staining scores and mast cell density were determined as previously described [37]. Briefly, the staining percentage of the relative number of cells stained was graded as follows: 0 for 0%, 1 for ≤ 25%, 2 for 25–50%, 3 for 50–75% and 4 for ≥ 75%. IHC intensity was scored as follows: 0 for no staining, 1 for weakly positive staining, 2 for moderately positive staining and 3 for strongly positive staining. The total score of each section was calculated by multiplying the intensity and percentage scores. For mast cell density (MCD), the number of mast cells in 5 random field (1 mM × 1 mM) was counted, and the average was MCD.

### Statistical analysis

Bioinformatics analyses were performed using the R software version 3.6.1. The Graph Pad Prism version 6.0 software (GraphPad, USA) was used to perform Pearson’s correlation analysis, linear regression analyses, and analyze differences between groups using Student’s t-test. A p value less than 0.05 was considered statistically significant.

## Supplementary Material

Supplementary Figures
