# Supplementary material for: CCL5-dependent mast cell infiltration into the tumor microenvironment in clear cell renal cell carcinoma patients
Source: Aging (Albany NY). 2020 Nov 11;12(21):21809–36. doi: 10.18632/aging.103999 (PMC7695370; doi:10.18632/aging.103999)
Supplement: Supplementary Figures [file aging-12-103999-s001..pdf]

SUPPLEMENTARY FIGURES

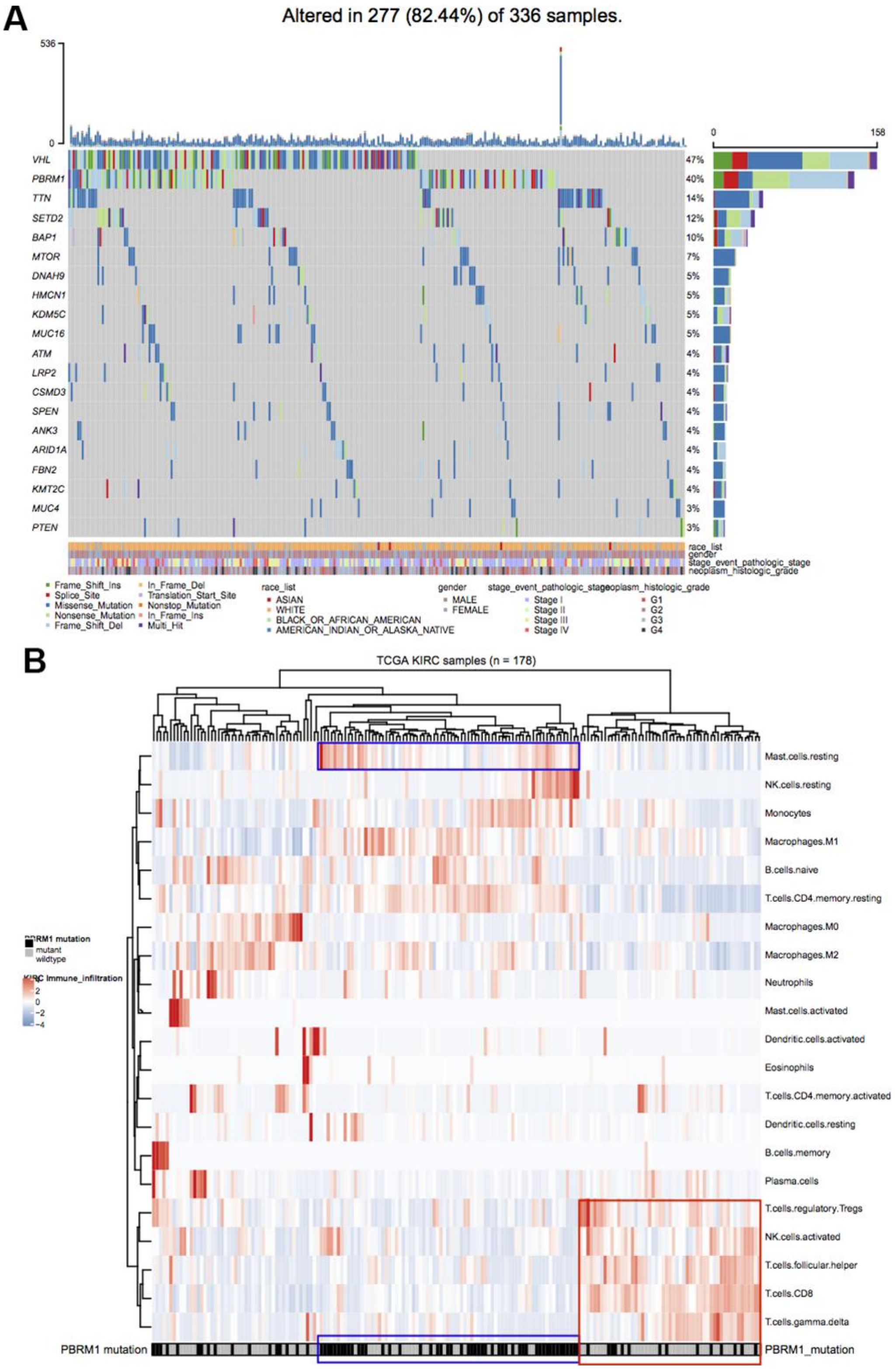

Supplementary Figure 1. Mutational signatures and immune landscape of ccRCC. (**A**) Genomic landscape of ccRCC (n=336) and the corresponding clinicopathological information of the TCGA KIRC dataset. (**B**) Twenty-two different types of immune cells across the distribution of 178 ccRCC samples in PBRM1^{WT} and PBRM1^{MUT}.

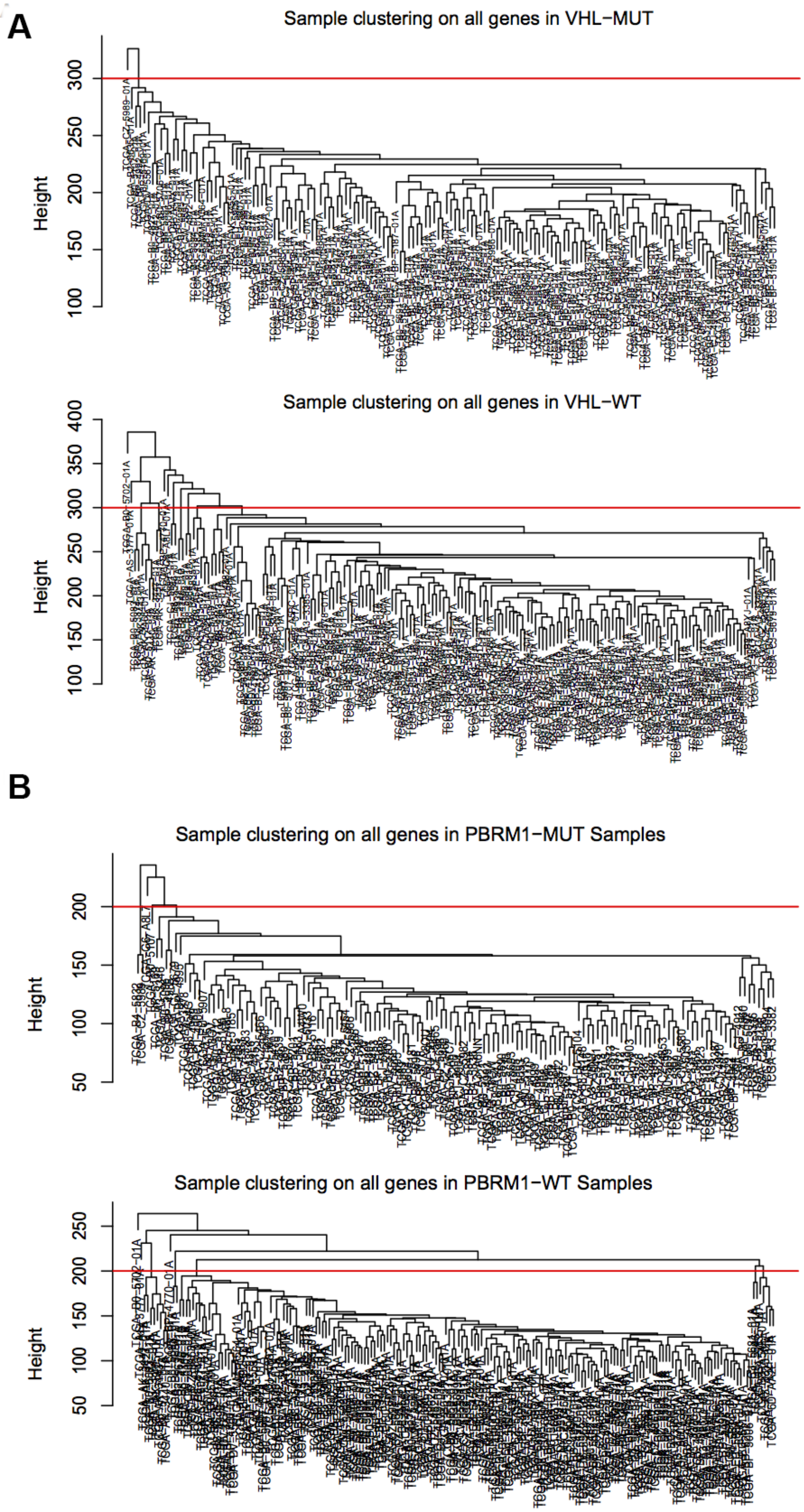

Supplementary Figure 2. Sample heterogeneity screening. (**A**, **B**) Dendrogram shows differentially expressed genes in VHL^{MUT} (177), VHL^{WT} (155), PBRM1^{MUT} (197) and PBRM1^{WT} (135) ccRCC samples. The red lines in the dendrograms represent cutoffs and samples outside these lines are considered outliers.

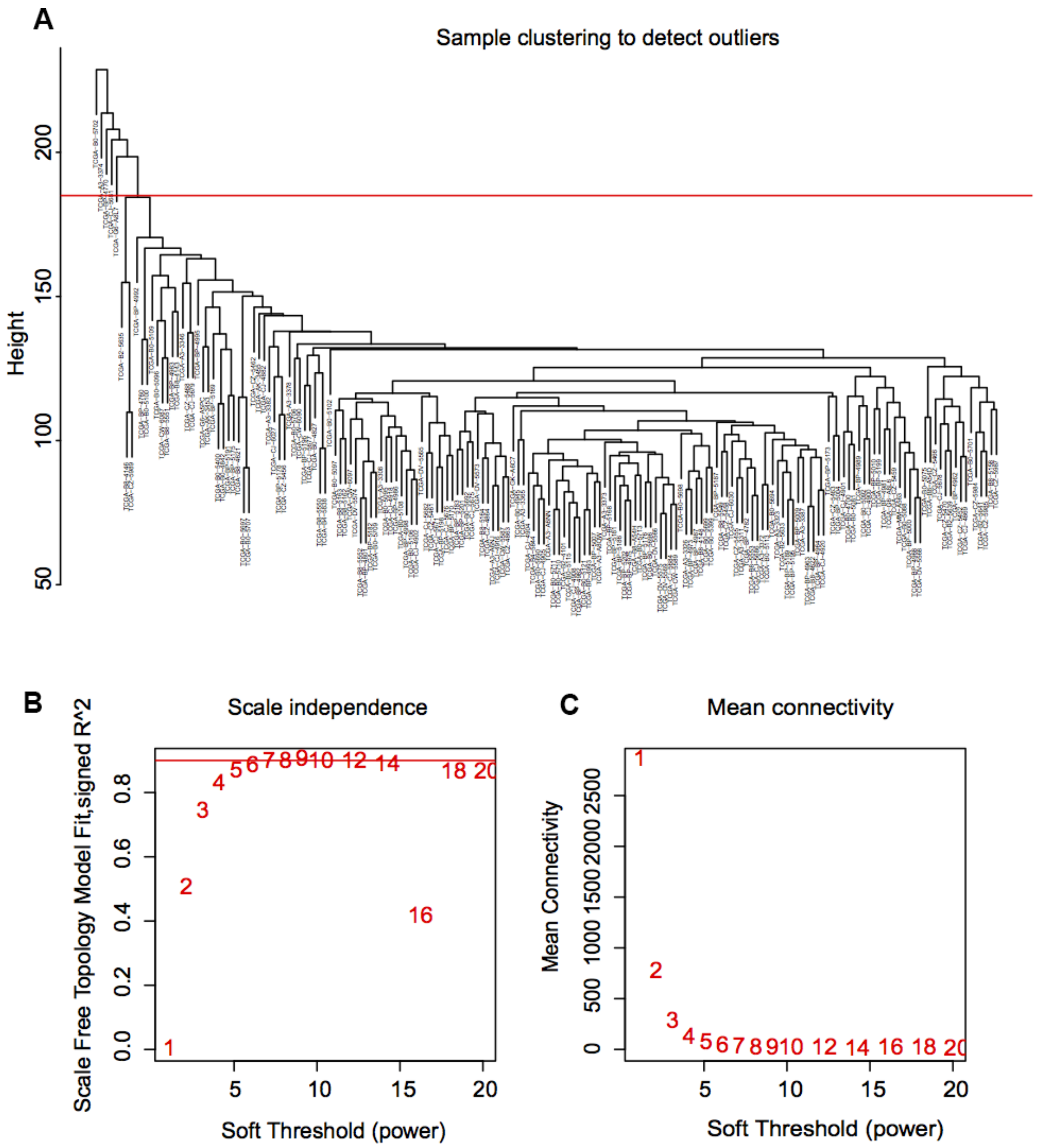

Supplementary Figure 3. Sample quality control. (**A**) Clustering dendrogram of ccRCC samples based on their Euclidean distance. (**B**, **C**) Analysis of the network topology for various soft-thresholding powers. The left panel shows the scale-free fit index (y-axis) as a function of the soft-thresholding power (x-axis). The right panel displays the mean connectivity (degree, y-axis) as a function of the soft-thresholding power (x-axis).

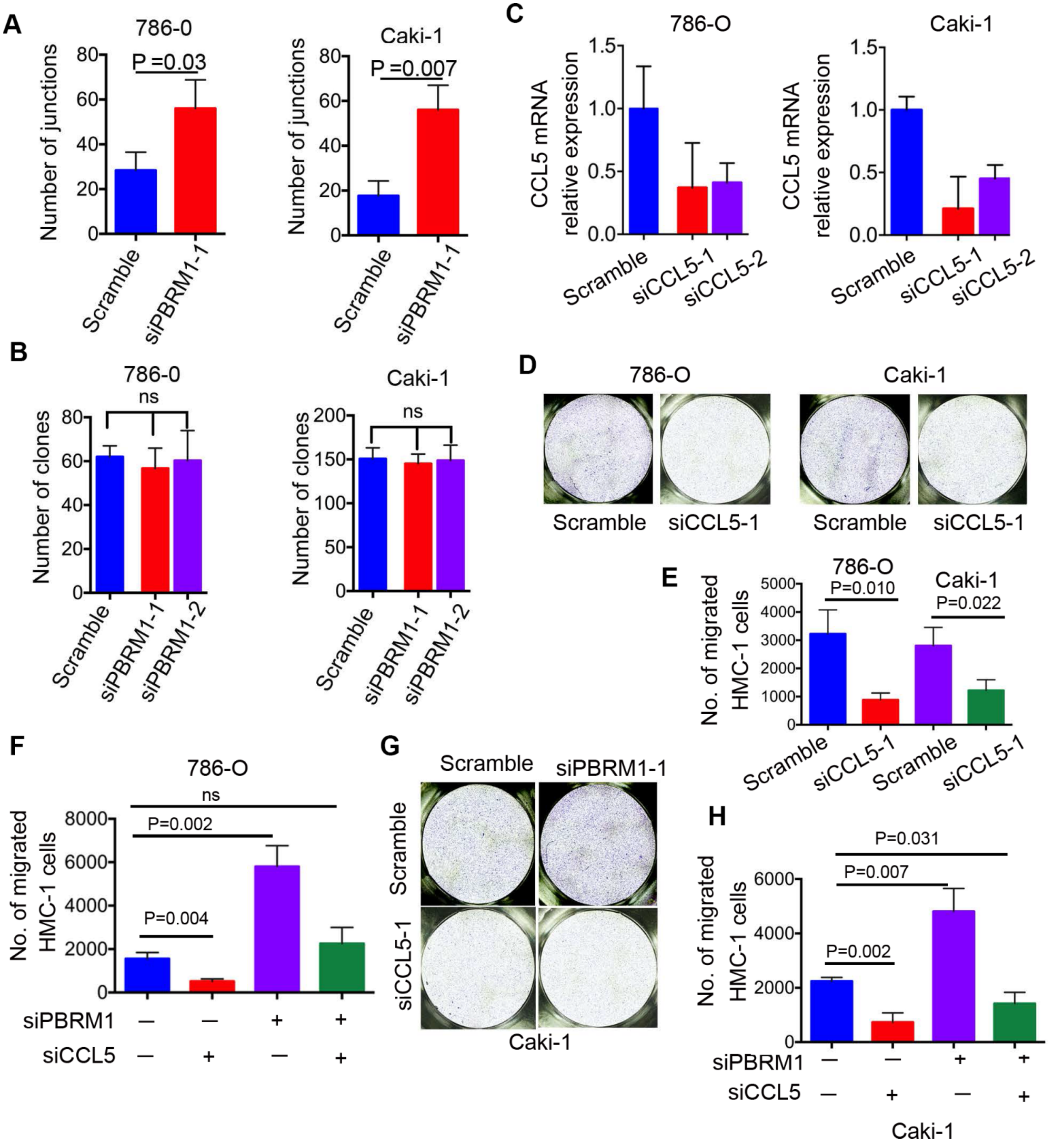

Supplementary Figure 4. The bio-function of PBRM1 protein in ccRCC cells. (**A**) The tube formation ability of control and PBRM1-silenced 786-O- and Caki-1 cells is shown. (**B**) Colony formation assay shows the number of colonies formed by control and PBRM1-silenced 786-O and Caki-1 cells. (**C**) qRT-PCR analysis shows CCL5 mRNA expression in control and siCCL5-transfected 786-O and Caki-1 cells, respectively. (**D**, **E**) Transwell migration assay shows migration ability of HMC-1 cells when co-cultured with CCL5-silenced in 786-O and Caki-1 cells, respectively. Note: All experiments were performed in triplicate; data was analyzed by Student's t-test and expressed as means ±SD; ns denotes not statistically significant. (**F**–**H**) Transwell migration assay shows migration ability of HMC-1 cells when co-cultured with PBRM1-silenced and PBRM1-silenced plus CCL5-silenced in 786-O and Caki-1 cells, respectively. Note: All experiments were performed in triplicate; data was analyzed by Student's t-test and expressed as means ±SD; ns denotes not statistically significant.

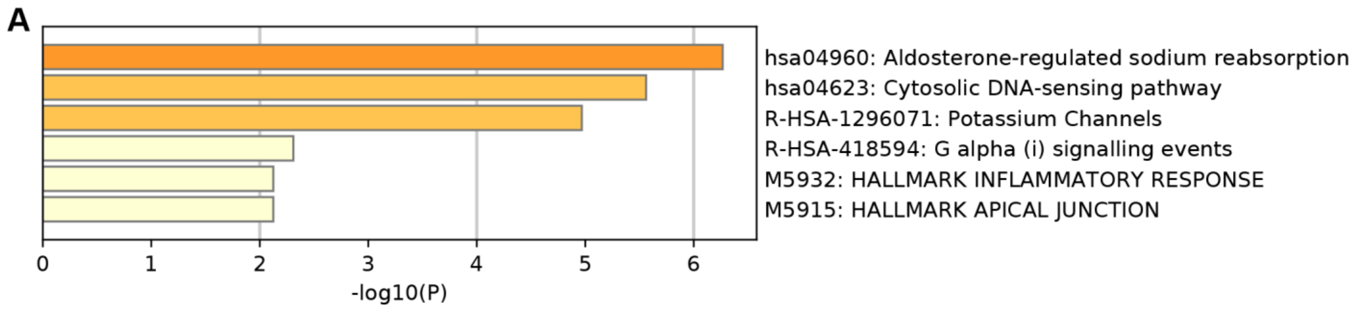

Supplementary Figure 5. The inflammatory response signaling pathway is suppressed in PBRM1-overexpressing ccRCC cells. (**A**) Pathway enrichment analysis of differentially expressed genes (DEGs) between control and PBRM1-overexpressing Caki-2 cells in the GSE76199 dataset.

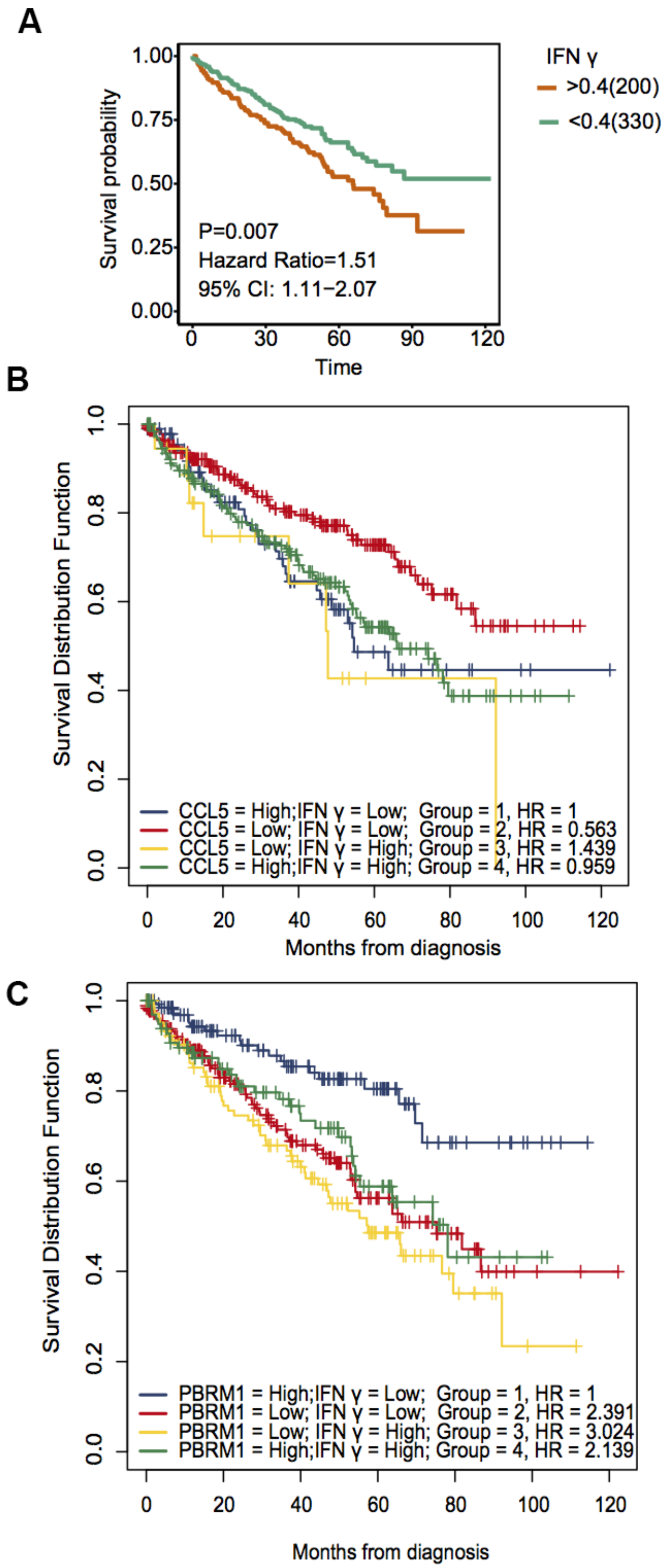

Supplementary Figure 6. High IFN-γ expression predicts worse overall survival in ccRCC patients from the TCGA KIRC database. (**A**) Survival analysis using survival and survminer packages shows overall survival of ccRCC patients with high (200) and low IFN-γ (330) expression. $P<0.05$ was considered statistically significant. (**B**) Survival analysis shows overall survival rates and HR values of ccRCC patients in the TCGA KIRC database, classified based on high and low CCL5 and IFN-γ expression. (**C**) Survival analysis shows overall survival rates and HR values of ccRCC patients in the TCGA KIRC database, classified based on high and low PBRM1 and IFN-γ expression.

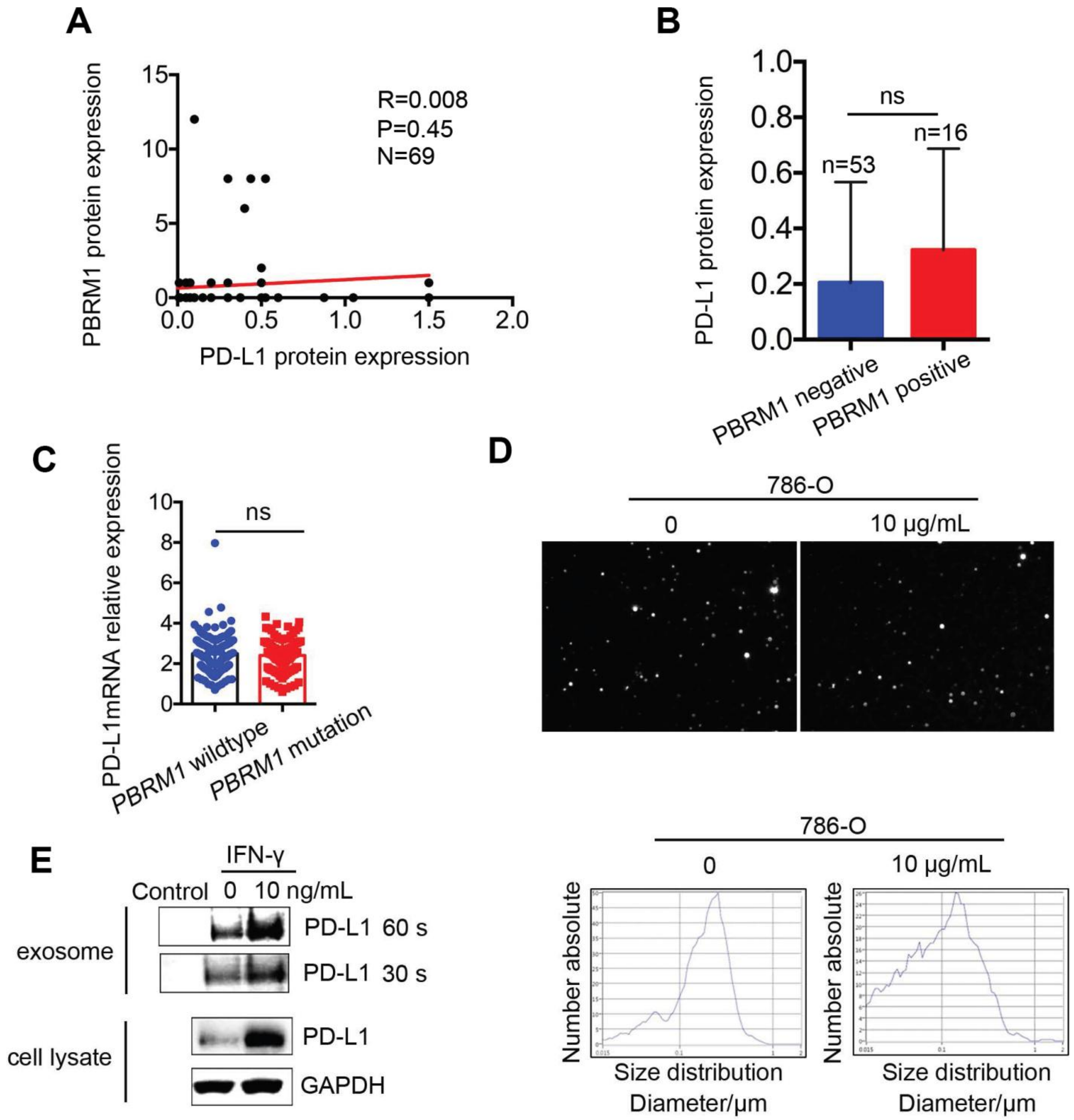

Supplementary Figure 7. PBRM1 expression does not correlate with PD-L1 expression in ccRCC. (**A**) The correlation analysis of PD-L1 IHC staining score and PBRM1 IHC staining score in ccRCC tissues. (**B**) PD-L1 mRNA expression in PBRM1-negative and PBRM1-positive ccRCC patients. (**C**) PD-L1 expression in $PBRM1^{WT}$ and $PBRM1^{MUT}$ ccRCC patients in the TCGA KIRC dataset. "ns" denotes 'not statistically significant'. (**D**) Characterization of exosomes purified from conditioned media of 786-O cells. Exosomes were identified by Zeta View, and the video data is included in the supplementary data. (**E**) Western blot analysis shows exosomal and total cellular PD-L1 protein levels in control and 10 ng/μL IFN-γ-treated 786-O cells and their corresponding. The loading buffer is loaded as "Control". '30 s' and '60 s' denote exposure time of 30 and 60 seconds, respectively.
